# Development of Imaging Complexity Biomarkers for Prediction of Symptomatic Radiation Pneumonitis in Patients with Non-Small Cell Lung Cancer, Focusing on Underlying Lung Disease

**DOI:** 10.3390/life14111497

**Published:** 2024-11-17

**Authors:** Jeongeun Hwang, Hakyoung Kim, Joon-Young Moon, Sun Myung Kim, Dae Sik Yang

**Affiliations:** 1 Department of Medical IT Engineering, College of Medical Sciences, Soonchunhyang University, Asan-si 31538, Republic of Korea; hwangje02@sch.ac.kr; 2 Departments of Radiation Oncology, Korea University Guro Hospital, Korea University College of Medicine, Seoul 08308, Republic of Korea; sunmyung01@hanmail.net; 3 Center for Neuroscience Imaging Research, Institute for Basic Science (IBS), Suwon-si 16419, Republic of Korea; joon.young.moon@gmail.com; 4 Sungkyunkwan University (SKKU), Suwon-si 16419, Republic of Korea

**Keywords:** lung cancer, radiotherapy, radiation pneumonitis, biomarker, lung disease

## Abstract

**Objectives:** We aimed to develop imaging biomarkers to predict radiation pneumonitis (RP) in non-small cell lung cancer (NSCLC) patients undergoing thoracic radiotherapy. We hypothesized that measuring morphometric complexity in the lung using simulation computed tomography may provide objective imaging biomarkers for lung parenchyma integrity, potentially forecasting the risk of RP. **Materials and Methods:** A retrospective study was performed on medical records of 175 patients diagnosed with NSCLC who had received thoracic radiotherapy. Three indices were utilized to measure the morphometric complexity of the lung parenchyma: box-counting fractal dimension, lacunarity, and minimum spanning tree (MST) fractal dimension. Patients were dichotomized into two groups at median values. Cox proportional hazard models were constructed to estimate the hazard ratios for grade ≥ 2 or grade ≥ 3 RP. **Results and Conclusions:** We found significant associations between lung parenchymal morphometric complexity and RP incidence. In univariate Cox-proportional hazard analysis, patients with a lower MST fractal dimension had a significantly higher hazard ratio of 2.296 (95% CI: 1.348–3.910) for grade ≥ 2 RP. When adjusted for age, sex, smoking status, category of the underlying lung disease, category of radiotherapy technique, clinical stage, histology, and DLCO, patients with a lower MST fractal dimension showed a significantly higher hazard ratio of 3.292 (95% CI: 1.722–6.294) for grade ≥ 2 RP and 7.952 (95% CI: 1.722 36.733) for grade ≥ 3 RP than those with a higher MST fractal dimension. Patients with lower lacunarity exhibited a significantly lower hazard ratio of 0.091 (95% CI: 0.015–0.573) for grade ≥ 3 RP in the adjusted model. We speculated that the lung tissue integrity is captured by morphometric complexity measures, particularly by the MST fractal dimension. We suggest the MST fractal dimension as an imaging biomarker for predicting the occurrence of symptomatic RP after thoracic radiotherapy.

## 1. Introduction

Radiation pneumonitis (RP) is a chronic side effect known to occur typically within 3 to 4 months following radiation therapy to thoracic organs. It begins as an inflammatory response in the lungs, and in certain instances, this can result in lasting scarring of lung tissue, referred to as pulmonary fibrosis. Additionally, in some situations, RP by itself may influence the morbidity and mortality of lung cancer patients.

Identifying and assessing the risk factors of severe RP in patients planning radiotherapy would be beneficial in clinical practice. There are several previous studies on various clinical factors that affect the development of severe RP [1,2,3,4,5], but a clear consensus has not yet been reached. Among these, there are prior studies showing that underlying lung disease can affect the development of severe RP [6,7,8,9,10,11,12,13,14,15]. Previous studies have indicated that patients with interstitial lung disease (ILD) are more prone to developing severe RP following high-dose radiotherapy [6,7,8]. It is important to note that ILD encompasses a wide range of conditions primarily affecting the lung parenchyma. Specifically, idiopathic pulmonary fibrosis (IPF) shows a higher rate of RP occurrence compared to other conditions [6,7,16,17], which can lead to a rapid worsening of the disease after radiation therapy, resulting in a poor prognosis. Conversely, the impact of chronic obstructive pulmonary disease (COPD) on the development of severe RP remains unclear [9,10,11]. As such, not just the categorical information on the existence of underlying pulmonary diseases but more objective, quantitative indices representing lung parenchymal integrity are desired to better assess a patient’s risk of RP.

Herein, we suggest that the measurement of morphometric complexity in the lung utilizing simulation computed tomography (CT) may provide objective, quantitative, and tractable imaging biomarkers for the lung parenchymal integrity that may foresee the risk of RP during radiotherapy. Indeed, a previous study [18] showed that patients with COPD presented with a lower box-counting fractal dimension, implying that poorer space-filling properties of lung parenchyma had worse survival outcomes. Whether the associations found in patients with COPD also apply to non-small cell lung cancer (NSCLC) patients with ILD undergoing radiotherapy is yet to be determined. Also, imaging biomarkers for morphometric complexity other than box-counting fractal dimension may also be explored for clinical utility. Extending the associations between the lower morphometric complexity in the lung parenchyma and poor prognosis found in COPD patients to NSCLC patients with ILD, we assume that a lower risk of severe RP may be expected in patients with higher parenchymal integrity in the lung, who would have higher morphometric complexity. In addition, our research team published preliminary results this year in the same journal, focusing on a small group of 19 patients diagnosed with idiopathic pulmonary fibrosis (IPF) [19]. Based on the positive findings from this initial study, we conducted further research to broaden the study to include other lung diseases.

In this context, our objective was to identify multifaceted risk factors, with a particular focus on the development of imaging complexity biomarkers, in order to predict the occurrence of severe RP in patients diagnosed with NSCLC undergoing thoracic radiation therapy.

## 2. Materials and Methods

### 2.1. Patients

Following the approval number 2023GR0216 from the Institutional Review Board, we retrospectively analyzed the medical records of 175 individuals diagnosed with NSCLC who underwent thoracic X-ray radiation therapy at Korea University Guro Hospital from June 2019 to June 2022. Due to the retrospective nature of this study, the Institutional Review Board waived the requirement for obtaining signed informed consent. Exclusion criteria involved patients who failed to finish radiation therapy or lacked pulmonary function test (PFT) data. The confirmation of underlying lung diseases was carried out by skilled pulmonologists.

### 2.2. Treatment Scheme and Surveillance

Following the institutional protocol, NSCLC patients with small-sized tumors (≤4 cm) situated peripherally were treated with stereotactic ablative radiation therapy (SABR), involving a total dose of 60 Gy administered in four fractions. For patients receiving intensity-modulated radiation therapy (IMRT), two dose-fractionation schedules were implemented: 60 Gy in 20 fractions for those in the radiotherapy-only group and 66/60 Gy in 30 fractions for patients receiving concurrent chemoradiotherapy, using the simultaneous integrated boost technique. 

Evaluation of treatment-related complications was carried out utilizing the Common Terminology Criteria for Adverse Events (version 5.0). In this study, grade 2 RP was defined as a case requiring outpatient steroid prescription, and grade 3 RP was defined as a severe side effect requiring hospitalization and oxygen treatment.

### 2.3. Measurement of Morphometric Complexity

For the quantification of the morphometric complexity of the lung parenchyma, three indices were adopted: a box-counting fractal dimension, a lacunarity, and a minimum spanning tree (MST) fractal dimension. We assessed pre-radiotherapy-simulated chest CT scans. The patients were scanned by Aquilion Lightning 80 (Cannon Medical Systems, Otawara, Japan), without contrast agent, and reconstructed to 1.0 × 1.0 × 2.5 mm voxel spacing DICOM format with a soft kernel. Detailed methods for assessing a box-counting fractal dimension and lacunarity are described in a previously published paper [19]. In brief, binary masks of intact lung parenchyma were defined as Normal Attenuation Areas (NAAs) at >−950 HU and ≤−700 HU. MATLAB (release 2023a, MathWorks Inc., Natick, MA, USA) scripts were built to measure the three indices including box-counting fractal dimension, lacunarity, and MST fractal dimension. The theoretical basis of a box-counting fractal analysis can be found in previous works by Grassberger [20] and Ott et al. [21]. A higher box-counting fractal dimension implies greater space-filling property [20,21]. A box-counting fractal dimension is estimated according to the power-law exponent, as in Formula (1):

(1) $${FD}_{box}\left(M\right)\propto \frac{\log N(\varepsilon )}{\log\left(\frac{1}{\varepsilon }\right)}$$

where *M* is the 3D mask of the NAA, *ε* is the cube size, and *N*(*ε*) is the number of size ε cubes needed to cover the mask.

Lacunarity is a measure of spatial heterogeneity and rotational invariance [22]. A lacunarity Λ is calculated from the probability density of pixels belonging to the 3D mask, as in Formula (2):

(2) $$\Lambda ={\left(\frac{\sigma_{\varepsilon ,\;g}}{\mu_{\varepsilon ,\;g}}\right)}^{2}$$

where *σ_ε,g_* is for the standard deviation of box-size ε and orientation *g*, and *μ_ε,g_* is for the average [22]. A higher Λ denotes higher rotational variance and a more heterogeneous spatial distribution [22].

An MST fractal analysis operates by creating a tree that spans all the NAA voxels without any loops and minimizes the total number of edges on the tree. While a box-counting fractal dimension represents the space-filling property of the NAA, an MST fractal dimension focuses on the connectivity and structure of the NAA. In general, MST fractal dimension is more suitable for datasets that exhibit a high degree of connectivity or network-like structures and may be more robust to the quantization error than a box-counting fractal dimension. In the field of complex dynamic systems, an MST fractal dimension method is used to compute the fractal dimensions of strange attractors [23,24], and then the relationship between the MST and fractal dimension has been proved rigorously [22]. We apply the MST method in the following steps. Given *N* NAA voxels, we choose *N_R_* nodes (voxels) randomly. The distance between two chosen nodes is the shortest path between two nodes (voxels) in the original NAA voxel data. The MST of the *N_R_* chosen nodes will be formed with *m = N_R_* − 1 edges and will have total distance of $\sum_{i=1}^{m}l_{i}$ where $l_{i}$ is the distance between chosen nodes. We can now form the following sum:

(3) $$S\left(m\right)=\frac{1}{m}\sum_{i=1}^{m}l_{i}m$$

This sum has a scaling behavior, as *m* is varied:

(4) $$S\left(m\right)=C\;m^{-\alpha /h}$$

Here, *C* is a constant, and *h* is calculated from this relation to yield an approximation to the fractal dimension. The h is called MST fractal dimension, and it is considered to give better approximation to the true dimension than the box-counting fractal dimension often biased to smaller approximations than the true value [23,24].

Taking logarithm of Formulas (3) and (4) yields Formula (5).

(5) $$\log\frac{1}{m}\sum_{i=1}^{m}l_{i}m\;\sim \;\frac{1}{h}\log m$$

The MST fractal dimension *h* is calculated with the linear regression on the basis of Formula (5).

### 2.4. Statistical Analyses

Categorical variables are presented as frequencies and percentages, while continuous variables are shown as median in tables. Time-to-event data on the occurrence of severe RP were used. The three morphometric complexity indices are stratified at median values to yield higher and lower strata. We built Cox proportional hazard models that were utilized to assess the hazards of morphometric complexity measures for RP. Hazard ratios, 95% confidence intervals, and Harrell’s C-indices were assessed by adjusting for age, gender, smoking status, category of the underlying lung disease, category of radiotherapy technique, clinical stage, and histology or not. Proportional hazard assumptions were attested by chi-square tests for the Shoenfeld residuals. When comparing continuous variables, a Student’s *t*-test was conducted after confirming equality of variances using Levene’s test and normality using the Shapiro–Wilk test. Multivariate generalized linear regression adjusting for age, gender, smoking status, category of the underlying lung disease, category of radiotherapy technique, clinical stage, and histology were performed to investigate associations. The threshold for statistical significance was established at *p* < 0.05. A sample size of at least 93 was calculated to ensure a power of 80% to detect a hazard ratio of 1.5 with a significance level of 0.05. All statistical analyses were conducted using R statistics software version 4.3.2 (R Foundation for Statistical Computing, Vienna, Austria).

## 3. Results

### 3.1. Baseline Characteristics

The characteristics of the patients are summarized in Table 1. The median age of the study population was 76 years old, and the age ranged from 38 to 93 years. The majority of the patients were male (76.0%), and a significant proportion were either current or former smokers (53.7%). Among the subjects, 51 patients (29.1%) were diagnosed with COPD, and 15 patients (8.6%) had IPF. Out of the 175 patients, 93 (53.1%) were diagnosed with stage I or II cancer, while the remaining 82 (46.9%) had stage III cancer. A total of 36 patients underwent SABR (20.6%), and 139 patients were treated with IMRT (79.4%).

### 3.2. Clinical Risk Factor Analysis

Table 2 provides the clinical characteristics, encompassing patient, tumor, and treatment-related factors (both chemotherapy and radiotherapy), categorized by the occurrence of severe RP. There were no statistically significant differences observed in variables like sex, smoking status, histology, or pretreatment pulmonary function test (PFT) values. Regarding radiotherapy planning parameters, factors like tumor volume and lung metrics (including MLD, V5, V10, and V20) were comparable between the groups. However, the presence of idiopathic pulmonary fibrosis (IPF) as a pre-existing lung condition (*p* < 0.001) and the clinical stage, influenced by concurrent chemotherapy use (*p* = 0.009), were associated with a higher risk of severe RP.

In the multivariate analysis, the presence of IPF as an underlying pulmonary condition showed a significant association with severe RP, and this association persisted following the application of the backward elimination method, as indicated in Table 3.

### 3.3. Morphometric Complexity Analysis

Box-counting fractal dimension, lacunarity, and MST fractal dimension analyses were conducted on pre-radiotherapy chest CT scans of enrolled patients. Patients were dichotomized at median values. The hazard ratios of lower-than-median measurements for grade ≥ 2 RP or grade ≥ 3 RP were assessed by building Cox proportional hazard models with adjusting and without adjusting. The adjusted models included age, sex, smoking status, category of the underlying lung disease, category of radiotherapy technique, clinical stage, histology, and DLCO. No significant violation of the proportional hazard assumption was found in the chi-square tests for the Schoenfeld residuals of each model. Table 4 shows the hazard ratios, 95% confidence intervals, *p*-values, and C-indices. Without adjustments, patients with a lower MST fractal dimension had a higher hazard ratio of 2.296 (95% CI: 1.348–3.910) for grade ≥ 2 RP. When adjusted, patients with a lower MST fractal dimension exhibited a significantly higher hazard ratio of 3.292 (95% CI: 1.722–6.294) for grade ≥ 2 RP and 7.952 (95% CI: 1.722 36.733) for grade ≥ 3 RP than those with a higher MST fractal dimension. Patients with lower lacunarity in their chest CT scans showed a significantly lower hazard ratio of 0.091 (95% CI: 0.015–0.573) for grade ≥ 3 RP in the adjusted model.

**Figure 1:**
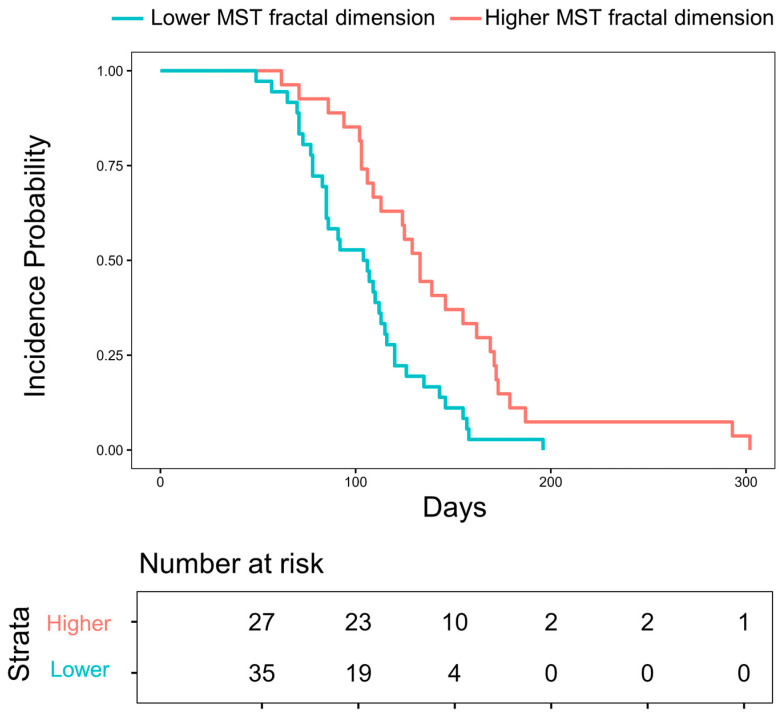
Kaplan–Meier curves for ≥grade 2 RP incidence in patients with higher (2.716–2.833) and lower (2.534–2.716) MST fractal dimension strata. The *p*-value of a log-rank test is 0.002.

Figure 1 shows Kaplan–Meier curves for grade ≥ 2 RP events in lower-than-median MST fractal dimension strata (blue) and higher-than-median strata (red). The lower-than-median MST fractal dimension group showed significantly steeper incidence curves with a *p*-value of 0.002 in a log-rank test. On the contrary, little significance was found in the log-rank tests for the Kaplan–Meier curves stratified in terms of box-counting fractal dimensions or lacunarity: *p*-values of 0.80 and 0.20, respectively, and provided in Supplementary Figure S1.

## 4. Discussion

Severe RP is among the most common treatment-related complications following high-dose lung radiotherapy and can influence the mortality rate in lung cancer patients. Although numerous studies have examined various factors contributing to severe RP [1,2,3], a consensus has yet to be reached. Additionally, the technical aspects of radiotherapy based on the latest advancements may not always be fully incorporated. Known risk factors for severe RP include (1) patient factors such as male sex, smoking history, underlying lung disease, and poor lung function; (2) tumor factors like tumor size and location; (3) treatment factors, including concurrent chemotherapy; and (4) dose–volume histogram-based dosimetric parameters like V5, V20, and mean lung dose (MLD), which are considered predictive markers for severe RP, though some debate remains.

This study aimed to identify different prognostic factors that may affect the development of severe RP in patients with NSCLC receiving high-dose radiotherapy, with or without concurrent chemotherapy. Clinical characteristics were classified into patient-related, tumor-related, and treatment-related factors. In Table 2 and Table 3, no statistically significant differences were observed in gender, smoking status, histology, or pretreatment PFT values. Regarding the radiotherapy plan parameters, although the tumor volume was slightly larger in the severe RP group (198.7 cm^3^ vs. 300.8 cm^3^, *p* = 0.011), the dosimetric parameters were similar between the groups. However, IPF as an underlying lung condition (*p* < 0.001) and the concurrent use of chemotherapy (*p* = 0.009) were associated with a higher incidence of severe RP, which aligns with prior findings. Analyzing specific underlying lung diseases, the incidence of severe RP was similar between the control and COPD groups in both the early-stage (3.2% vs. 4.3%) and locally advanced-stage (15.2% vs. 10.7%) NSCLC. In contrast, IPF was strongly associated with the development of severe RP across all stages of NSCLC, with incidences of 42.9% in the early stage and 75.0% in the locally advanced stage.

We found significant associations between lung parenchymal morphometric complexity and RP incidence. In the previous study [18] performed on COPD patients, a box-counting fractal dimension in the lung’s NAA was associated with survival outcomes. Emphysema in COPD is a deleterious disease, so a lower box-counting fractal dimension representing lower space-filling power of the NAA would imply severer emphysema and poorer lung tissue integrity, which may lead to a worse prognosis. Similarly, in the current study, we speculated that ILD patients with poorer lung tissue integrity would exhibit lower morphometric complexity in NAA, thereby resulting in higher susceptibility to RP. Unlike emphysema, ILD is a multifaceted disease that is not only deleterious but includes abnormal CT findings such as fibrosis, consolidation, honeycombing, ground glass opacity, air trapping, and emphysema [25]. These multifaceted CT anomalies alter the lung tissue’s X-ray attenuation to both upward and downward. For example, anomalies including emphysema and air trapping would lower the attenuation value under −950 HU, while consolidation, fibrosis, and ground glass opacity may enhance the attenuation. These abnormalities share a common characteristic: They simplify the complex structures of the normal lung parenchyma. So, we binarized the NAA by applying a threshold of −950 HU to −700 HU and underwent morphometric complexity analysis.

A box-counting fractal dimension measures the space-filling property, a lacunarity assesses rotational invariance, and an MST fractal dimension quantifies structural complexity. Among the three morphometric complexity indices, the MST fractal dimension showed the most robust associations with RP outcomes. In Table 4, the MST fractal dimension has significant associations with grade ≥ 2 RP both in the unadjusted and adjusted models and with grade 3 ≥ RP in the adjusted model. Considering that an MST fractal dimension focuses on the connectivity and structures of a graph, it is rational to understand that the lung tissue integrity would be better measured and represented by the MST fractal analysis beyond a simple space-filling property or rotational invariance. Also, the MST fractal method is known to be a better approximator for the true value of dimension [22,23,24], and this may have contributed to the MST fractal dimension being better associated with the RP outcomes. Accordingly, in Figure 1, patients with a higher MST fractal dimension in their NAAs showed a lower risk of severe RP compared to those with lower values, suggesting that the imaging biomarker could be a useful predictor of RP prognosis.

The current study has a limitation in that it is a single-center, retrospective study. Since we found a potential for RP prognosis in imaging biomarkers concerning morphometric complexity and lung parenchymal integrity, it highlights the need for further multi-center or prospective studies to validate these findings externally.

## 5. Conclusions

We showed a significant association between morphological complexity measures of NAA in simulated CT scans and RP incidence. Specifically, we suggest an MST fractal dimension as an imaging biomarker for predicting the occurrence of symptomatic RP after thoracic radiotherapy. Morphometric complexity, especially MST fractal dimension is warranted for further investigations in multi-centric, prospective settings for external validation, robustness analyses, and clinical utility analyses to be incorporated into the clinical workflow.

## Figures and Tables

**Table 1:** Characteristics of patients undergoing thoracic radiation therapy (N = 175).

Characteristics	Number	%
Age [years; median (range)]	76 (38–93)	
Gender		
Female	42	24.0%
Male	133	76.0%
Smoking status		
Never smoker	81	46.3%
Current or ex-smoker	94	53.7%
ECOG performance status		
0–1	152	86.9%
2	23	13.1%
Underlying lung diseases		
None	109	62.3%
COPD	51	29.1%
IPF	15	8.6%
Clinical stage		
I–II	93	53.1%
III	82	46.9%
Histology		
Squamous cell carcinoma	81	46.3%
Adenocarcinoma	88	50.3%
Others	6	3.4%
Radiotherapy technique		
Stereotactic ablative radiotherapy	36	20.6%
Intensity-modulated radiotherapy	139	79.4%

Abbreviations: ECOG, Eastern Cooperative Oncology Group; COPD, chronic obstructive pulmonary disease; IPF, idiopathic pulmonary fibrosis.

**Table 2:** Clinical characteristics based on the occurrence of severe RP (N = 175).

Characteristics	≤Grade 2 RP	≥Grade 3 RP	*p*-Value
Age [years; median (range)]	76 (38–93)	75 (65–87)	0.099
Gender			0.350
Female	38 (90.5%)	4 (9.5%)	
Male	115 (86.5%)	18 (13.5%)	
Smoking status			0.318
Never smoker	73 (90.1%)	8 (9.9%)	
Current or ex-smoker	80 (85.1%)	14 (14.9%)	
ECOG performance status			0.202
0–1	131 (86.2%)	21 (13.8%)	
2	22 (95.7%)	1 (4.3%)	
Underlying lung diseases			<0.001
None	100 (91.7%)	9 (8.3%)	
COPD	47 (92.2%)	4 (7.8%)	
IPF	6 (40.0%)	9 (60.0%)	
Clinical stage			0.009
I-II	87 (93.5%)	6 (6.5%)	
III	66 (80.5%)	16 (19.5%)	
Histology			0.637
Squamous cell carcinoma	69 (85.2%)	12 (14.8%)	
Adenocarcinoma	79 (89.8%)	9 (10.2%)	
Others	5 (83.3%)	1 (16.7%)	
Pulmonary function test (mean)			
FEV1	2.1L	2.1L	0.469
FEV1 % predicted	73.9%	80.9%	0.082
FVC	3.1L	2.9L	0.213
FVC % predicted	78.9%	77.4%	0.093
FEV1/FVC	64.7%	71.8%	0.533
DLco	70.4%	67.4%	0.274
Planning parameter	198.8		
Total lung_MLD	745.5 cGy	977.9 cGy	0.027
Total lung_V5	36.2%	47.7%	0.030
Total lung_V20	14.3%	16.7%	0.431

Abbreviations: RP, radiation pneumonitis; ECOG, Eastern Cooperative Oncology Group; COPD, chronic obstructive pulmonary disease; IPF, idiopathic pulmonary fibrosis; FEV1, forced expiratory volume in 1 s; FVC, forced vital capacity; DLCO, diffusing capacity of the lung for carbon monoxide; MLD, mean lung dose; VD, percentage volume of organ receiving ≥ D Gy. *p*-values: Student’s *t*-tests for continuous variables and chi-square tests for categorical variables.

**Table 3:** Associations between patient characteristics and the incidence of severe RP (≥Grade 3).

Characteristics	Severe RP	*p*-Value	Retained
Age	4.16 × 10^−2^ (−2.72 × 10^−2^–0.120)	0.265	
Male gender	0.931 (0.167–5.20)	0.934	
Current or ex-smoker	0.718 (0.181–2.97)	0.636	
Histology			
SqCC	0.753 (0.199–2.72)	0.668	
ADC	0.960 (0.042–8.68)	0.974	
Others			
Clinical stage III	3.69 (0.721–21.8)	0.129	Yes
Underlying lung diseases			
None			
COPD	1.32 (0.292–5.59)	0.706	
IPF	48.4 (9.09–347)	<0.001 *	Yes
DLco < 80%	0.264 (0.060–1.00)	0.056	Yes
Plan parameter			
Total lung_MLD	1.21 × 10^−3^	0.539	
	(−2.58 × 10^−3^–7.01× 10^−3^)		
Total lung_V5	−9.39 × 10^−3^	0.793	
	(−8.29 × 10^−2^–5.94 × 10^−2^)		
Total lung_V20	−1.27 × 10^−2^	0.674	
	(−3.49 × 10^−2^–9.45 × 10^−3^)		

Abbreviations: RP, radiation pneumonitis; SqCC, squamous cell carcinoma; ADC, adenocarcinoma; COPD, chronic obstructive pulmonary disease; IPF, idiopathic pulmonary fibrosis; DLCO, diffusing capacity of the lung for carbon monoxide; MLD, mean lung dose; VD, percentage volume of organ receiving ≥ D Gy. * Odds ratios of categorical variables and βs of continuous variables and their 95% confidence intervals for Grade 3 or higher radiation pneumonitis by multivariate generalized linear regression. Variables retaining after backward elimination are marked as ‘Yes’.

**Table 4:** Associations between risk factors and incidence of severe RP by using Cox’s proportional hazard model.

				HR ^b^ (95% CI ^c^)	C-Index ^d^	*p*-Value
Grade ≥ 2 (# of events = 63)	Unadjusted model	Box-counting fractal dimension	2.173–2.363 (N = 88)	Reference		
			1.956–2.173 (N = 87)	1.321 (0.786–2.220)	0.522	0.294
		Lacunarity	0.461–0.743 (N = 88)	Reference		
			0.317–0.456 (N = 87)	0.735 (0.438–1.234)	0.522	0.245
		MST fractal dimension	2.716–2.833 (N = 88)	Reference		
			2.534–2.716 (N = 87)	2.296 (1.348–3.910)	0.614	0.002
	Adjusted model ^a^	Box-counting fractal dimension	2.173–2.363 (N = 88)	Reference		
			1.956–2.173 (N = 87)	1.565 (0.841–2.911)	0.610	0.157
		Lacunarity	0.461–0.743 (N = 88)	Reference		
			0.317–0.456 (N = 87)	0.711 (0.387–1.307)	0.606	0.272
		MST fractal dimension	2.716–2.833 (N = 88)	Reference		
			2.534–2.716 (N = 87)	3.292 (1.722–6.294)	0.666	< 0.001
Grade ≥ 3 (# of events = 22)	Unadjusted model	Box-counting fractal dimension	2.173–2.363 (N = 88)	Reference		
			1.956–2.173 (N = 87)	0.892 (0.375–2.122)	0.480	0.796
		Lacunarity	0.461–0.743 (N = 88)	Reference		
			0.317–0.456 (N = 87)	1.126 (0.476–2.663)	0.491	0.788
		MST fractal dimension	2.716–2.833 (N = 88)	Reference		
			2.534–2.716 (N = 87)	1.614 (0.671–3.882)	0.609	0.285
	Adjusted model	Box-counting fractal dimension	2.173–2.363 (N = 88)	Reference		
			1.956–2.173 (N = 87)	1.024 (0.222–4.722)	0.678	0.976
		Lacunarity	0.461–0.743 (N = 88)	Reference		
			0.317–0.456 (N = 87)	0.091 (0.015–0.573)	0.776	0.002
		MST fractal dimension	2.716–2.833 (N = 88)	Reference		
			2.534–2.716 (N = 87)	7.952 (1.722–36.733)	0.803	0.008

^a^ Adjusted model: adjusted for age, gender, smoking status, category of the underlying lung disease, category of radiotherapy technique, clinical stage, histology, and DLCO%. ^b^ HR: hazard ratio. ^c^ CI: confidence interval. ^d^ C-index: Harrel’s C. Note: Box-counting fractal dimension, lacunarity, and MST fractal dimension are in real numbers without any unit.

## Data Availability

The datasets used and/or analyzed in the current study can be obtained from the corresponding author upon reasonable request.

## Supplementary Materials

The following supporting information can be downloaded at: https://www.mdpi.com/article/10.3390/life14111497/s1, Figure S1: Log-rank tests for the Kaplan-Meier curves stratified in terms of box-counting fractal dimensions or lacunarity: *p* values of 0.80 and 0.20, respectively.
