# Supplementary figures and images for: Development of Imaging Complexity Biomarkers for Prediction of Symptomatic Radiation Pneumonitis in Patients with Non-Small Cell Lung Cancer, Focusing on Underlying Lung Disease
Source: Life (Basel). 2024 Nov 17;14(11):1497. doi: 10.3390/life14111497 (PMC11595866; doi:10.3390/life14111497)

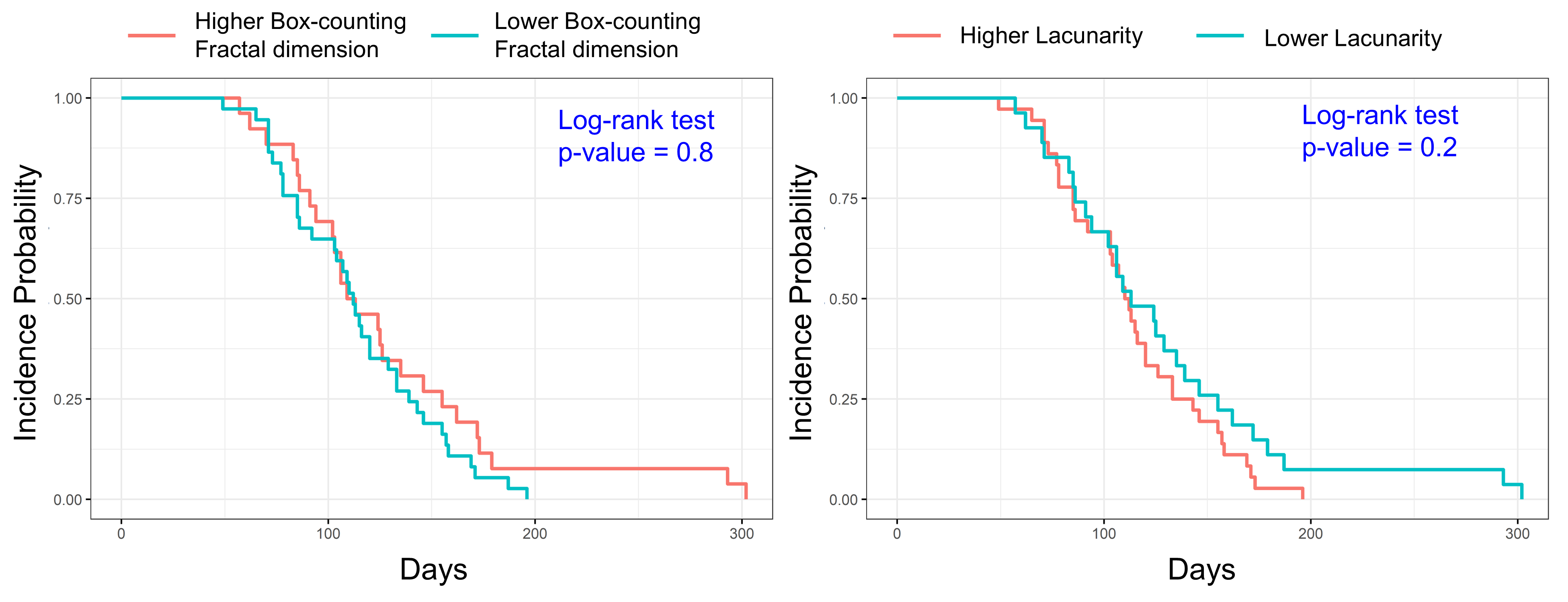

Supplement: Supplementary file 1 [file life-14-01497-s001.zip › life-3265293-supplementary.tif]
